# Prediction of PSA Response after Dexamethasone Switch during Abiraterone Acetate + Prednisolone Treatment of Metastatic Castration-Resistant Prostate Cancer Patients

**DOI:** 10.3390/cancers16152760

**Published:** 2024-08-03

**Authors:** Bertalan Fekete, Krisztina Biró, Fruzsina Gyergyay, Nándor Polk, Orsolya Horváth, Lajos Géczi, Attila Patócs, Barna Budai

**Affiliations:** 1 Department of Endocrinology and Diabetology, Internal Medicine II, Central Hospital of Northern Pest—Military Hospital, Podmaniczky u. 109–111, 1062 Budapest, Hungary; 2 Department of Genitourinary Medical Oncology and Clinical Pharmacology, National Institute of Oncology, Comprehensive Cancer Center, Ráth Gy. u. 7-9, 1122 Budapest, Hungary; 3 Department of Visceral Surgery, National Institute of Oncology, Comprehensive Cancer Center, Ráth Gy. u. 7-9, 1122 Budapest, Hungary; 4 National Tumor Biology Laboratory, National Institute of Oncology, Comprehensive Cancer Center, Ráth Gy. u. 7-9, 1122 Budapest, Hungary; 5 Department of Laboratory Medicine, Semmelweis University, Üllői út 78/b, 1083 Budapest, Hungary; 6 Department of Molecular Genetics, National Institute of Oncology, Comprehensive Cancer Center, Ráth Gy 7-9, 1122 Budapest, Hungary

**Keywords:** corticosteroid switch, predictive model, responders, treatment duration, overall survival

## Abstract

**Simple Summary:**

A model predicting responders to corticosteroid switch during abiraterone therapy in patients with metastatic castration-resistant prostate cancer is warranted. The model using logistic regression was developed in a cohort of 67 and validated in another cohort of 42 patients. The use of this model is easy and all needed parameters are available from routine laboratory and history of patients. The overall accuracy was 92%. Multivariate analysis revealed that responsiveness was a marker of longer treatment duration and overall survival. For non-responders a new systemic treatment was indicated.

**Abstract:**

Background: The aim was to elaborate a predictive model to find responders for the corticosteroid switch (from prednisolone to dexamethasone) at the first prostate-specific antigen (PSA) progression (≥25% increase) during abiraterone acetate (AA) treatment of metastatic castration-resistant prostate cancer (mCRPC) patients. Methods: If PSA has decreased (≥25%) after switch, patients were considered responders. Logistic regression of 19 dichotomized parameters from routine laboratory and patients’ history was used to find the best model in a cohort of 67 patients. The model was validated in another cohort of 42 patients. Results: The model provided 92.5% and 90.5% accuracy in the testing and the validation cohorts, respectively. Overall the accuracy was 91.7%. The AUC of ROC curve was 0.92 (95% CI 0.85–0.96). After a median follow-up of 27.9 (26.3–84) months, the median AA+dexamethasone treatment duration (TD) in non-responders and responders was 4.7 (3.1–6.5) and 11.1 (8.5–12.9) months and the median overall survival (OS) was 23.2 (15.6–25.8) and 33.5 (26.1–38) months, respectively. Multivariate Cox regression revealed that responsiveness was an independent marker of TD and OS. Conclusions: A high accuracy model was developed for mCRPC patients in predicting cases which might benefit from the switch. For non-responders, induction of the next systemic treatment is indicated.

## 1. Introduction

The incidence of prostate cancer is high both in the world and in our country [1]. Prostate cancer can reach a stage where it becomes castration-resistant and metastasizes (most commonly to bone, but also to lymph nodes and/or visceral sites). 

Within the systemic treatment modalities for metastatic castration-resistant prostate cancer (mCRPC), a growing number of drugs are becoming available that target tumor cells by different mechanisms [2]. One such drug is abiraterone acetate (AA). AA treatment inhibits androgen synthesis at the cellular level by blocking the CPY17A1 enzyme. In the absence of androgens, the levels of testosterone and dihydrotestosterone are reduced. In the absence of a ligand, the androgen receptor (AR), which is responsible for prostate cancer cell proliferation, is blocked. A common side effect of androgen synthesis inhibition is an increase in adrenocorticotropic hormone (ACTH) levels. This side effect can be prevented by the administration of corticosteroids. The use of prednisone/prednisolone is recommended according to the AA’s protocol. 

Resistance to AA can develop through different mechanisms. The first sign of resistance could be an increase in PSA levels. In this case, if there is no clinical or radiological progression, prednisolone can be switched to dexamethasone. After the switch, reduction or stabilization of PSA was observed in >56% of patients (>48% with a PSA decrease > 50%). [3]. 

The main question is: who will be a responder (PSA decreases) or non-responder (no change or increasing PSA) after the switch? To the best of our knowledge, this question has not been answered. Several markers of longer PSA progression-free survival (PPFS) after switching to dexamethasone have been investigated by several authors, but these results are inconsistent [4].

The aim of the study was to develop a predictive model to identify responders to corticosteroid switching (from prednisolone to dexamethasone) during AA treatment of mCRPC patients at first PSA progression (≥25% increase). This is also important because non-responders can be moved to the next line of systemic treatment to achieve longer overall survival (OS).

## 2. Patients and Methods

All consecutive patients with mCRPC treated at our National Institute Comprehensive Cancer Center who started AA treatment between 2013 March and 2022 September and switched from prednisolone to dexamethasone at the first PSA progression were enrolled. Inclusion criteria: first line AA treatment (pre-chemotherapy) or second line treatment (post-docetaxel). All patients were androgen receptor-targeted agent treatment-naïve. 

### 2.1. Treatment and Follow-Up

AA+prednisolone was administered according to the treatment protocol, including 1000 mg AA and 10 mg prednisolone daily. Prednisolone was changed to 0.5 mg dexamethasone when serum level of PSA increased and no other sign of progression was present [5]. The AA treatment was covered until 2 of the 3 progression signs (raising PSA, radiological or clinical progression) appeared. 

The castration resistance-free survival (CRFS) was calculated from the date of diagnosis to the beginning of AA or chemotherapy, based on which came first. The site of the metastases and ECOG performance status were recorded before AA treatment. The performance status was also registered at the time of the switch.

Follow-up of all patients was performed every 3 months. This included physical examination, abdominal ultrasonography, CT, MRI or bone scan, and laboratory evaluation of hematological parameters, liver and kidney functions and PSA determination. 

The PPFS was calculated from the start of AA to the date of first PSA increase (≥25%). AA treatment duration after switch (TD), overall survival (OS) calculated from the date of switch until death or last follow-up of patients, and the number of systemic treatment lines after AA were also recorded. 

Responders to corticosteroid switch were defined as such if PSA level decreased (≥25%) after the switch. All other patients were considered non-responders. Although in the literature a 30% (PSA30_D_) or 50% decrease of PSA (PSA50_D_) after switching was considered [4], we examined our TD and OS survival curves and found the largest difference between the survival curves at 25% PSA decrease (cut-off value). Among several authors who used this cut-off value, Walter et al. [6] defined minor response as ≥25–49% PSA decrease in mCRPC patients receiving capecitabine, rofecoxib, pioglitazone and dexamethasone. 

### 2.2. Laboratory Measurements

Blood tests were taken at the beginning of AA therapy. PSA and routine laboratory blood tests were performed at one and three months after the initiation of AA and every 1 or 3 months thereafter. Serum PSA level was determined using LIAISON^®^ PSA ILMA kit (DiaSorin S.p.A., Saluggia, Italy).

### 2.3. Statistics

The primary objective was the PSA response after the corticosteroid switch. The secondary objective was the TD and OS after switch.

The cut-off of parameters, except for Gleason score (Gl), were determined with the ROC analysis considering the response to dexamethasone.

For univariate analysis, t-test (or Mann–Whitney U-test) or chi2-test (or Fisher exact test) was used to compare responders and non-responders. Multivariate logistic regression was performed to find the best model to separate responders from non-responders. The significance level of parameters included in logistic regression was determined with a likelihood ratio test. In the search for the best model, dichotomized variables for which the *p* value was <0.2 in univariate analysis were selected first. All variables which caused non-convergence of the logistic regression model were omitted. After trial and error, additional covariates were included because they increased the accuracy of the model. The significance level was considered at *p* < 0.05. The elaboration of the model was performed in a subset of patients (61% of all patients were randomly selected). Validation was also carried out in a cohort including the other patients. A standard 5-fold cross-validation was also performed. Kaplan–Meier method, log rank test and multivariate Cox regression were used for analysis of TD and OS. Some parameters were omitted from multivariate Cox regression to avoid multicollinearity. The NCSS 2019 Statistical Software (NCSS, LLC. Kaysville, UT, USA) was used for statistical analyses.

## 3. Results

In total, 115 patients were enrolled and 70 and 45 were included in the test and validation cohorts, respectively. Due to certain laboratory parameters being missing from both groups, 3 to 3 patients were excluded. The best predictive model was constructed in a cohort of 67 patients and another cohort of 42 patients was used for validation. 

**Figure 1:**
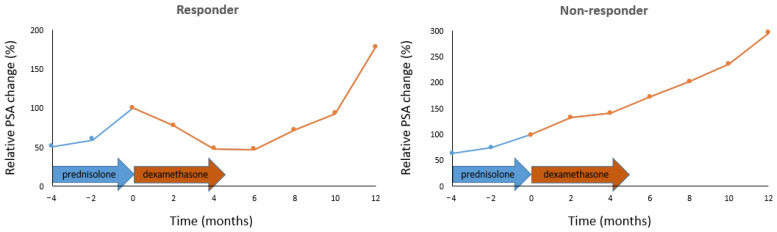
Change in PSA in responders and non-responders after switch during AA treatment of mCRPC patients. AA, abiraterone acetate; mCRPC, metastatic castration-resistant prostate cancer; PSA, prostate-specific antigen.

The representative PSA change of responders and non-responders is presented in Figure 1.

The characteristics of responders and non-responders are presented in Table 1.

In order to find the best predictive model, logistic regression analysis was performed on 67 patients (34 non-responders and 33 responders). The best model was obtained by including the following 19 dichotomized parameters in the multivariate logistic regression analysis: age, Gl, CRFS, pre, PPFS, visc, PSAs, PSAw, ALPw, LDHs, LDHw, hgbw, WBCs, WBCw, lys, lyw, neuw, pls and plw. The explicit form of the resulted model was the following: response or non-response (1 or 0, respectively) = 1/(1 + 10^−A^), where

A = −1.69 + 2.51 × age + 0.65 × Gl + 2.6 × CRFS + 1.58 × pre − 4.1 × PPFS + 0.95 × visc − 7.17 × PSAs + 1.17 × PSAw − 0.74 × ALPw − 1.5 × LDHs + 5.02 × LDHw + 2.48 × hgbw + 0.37 × WBCs − 0.84 × WBCw − 0.58 × lys + 1.78 × lyw + 2.48 × neuw − 2.82 × pls + 1.15 × plw).

If A > 0, then the tested patient is likely to be responder, and otherwise a non-responder. The likelihood ratio test revealed a very good fit of the model (*p* = 3.8 × 10^−4^) resulting in 92.5% correct classification (four responders were estimated as non-responders and 1 non-responder as responder).

Applying the above model on 42 patients (26 non-responders and 16 responders) as the validation cohort, 90.5% was correctly classified (4 non-responders were estimated as responders). If the testing and validation groups were merged, the correct classification (accuracy) was 91.74% (100/109). The precision and sensitivity were 98.11% and 86.67%, respectively. The area under the ROC curve was 0.92 (95% CI 0.85–0.96) and *p* = 2.5 × 10^−55^.

The mean accuracy of standard 5-fold cross-validation was 91.72% (95% CI 90.43–93). Knowing the level of the listed parameters for a given patient, Table 2 (and Supplementary Table S1) help to determine whether the patient will benefit from the AA+dexamethasone treatment. 

It can be seen from Table 2 that some parameters are superior in forming the total (PSA at start, PPFS, LDH at switch); furthermore, most of the relatively elevated laboratory parameters at the beginning are negative, while most of the relatively elevated laboratory parameters at switch have a beneficial effect.

**Figure 2:**
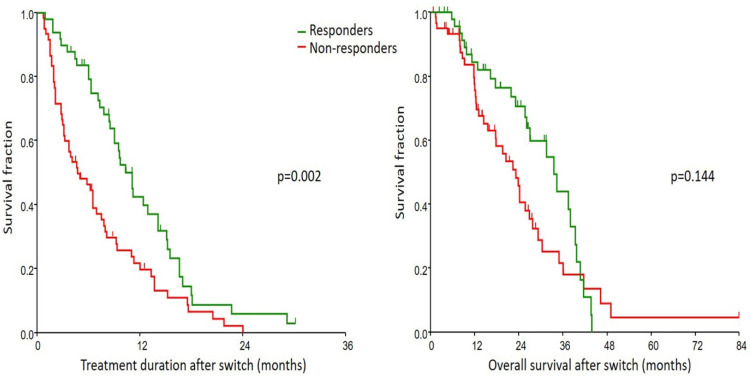
Post-switch AA treatment duration and overall survival according to PSA response after switch. AA, abiraterone acetate; PSA, prostate-specific antigen; responder, ≥25% decrease in PSA level after switch.

The median follow-up was of 27.9 (95% CI 26.3–84) months. TD and OS after switch are presented in Figure 2. (See Table 1 for median survival values).

Multivariate Cox regression (including parameters, which had *p* < 0.1 in univariate analysis of TD and which had *p* < 0.2 in univariate analysis of OS) revealed that responsiveness was a significant and independent marker of TD and OS (Table 3).

To avoid misuse of the model, five patients with hormone sensitive prostate cancer who received AA and were switched from prednisolone to dexamethasone at the first PSA progression was also tested and none were correctly classified. 

## 4. Discussion

Several authors investigated the possibility of switch-related prediction [3,5,7,8,9,10,11,12,13,14,15,16,17,18,19], but only 6 authors found predictive markers for PSA30_D_ [8,10,14,18] and PSA50_D_ [10,15,16]; the others reported predictive factors of PPFS. Arciero et al. [8], in a study of 11 patients, found that responders during prednisolone (PSA30) were three times more likely to be responders during dexamethasone (PSA30_D_), but the difference was not significant due to the small number of cases. The SWITCH study involving 26 patients reported by Romero-Laorden et al. [10] showed that patients with AR gain had significantly shorter PPFS and radiological PFS. Barua et al. [14] presented results of 87 patients and the favourable ECOG performance status and first-line AA were associated with PSA30_D_. Ni et al. [18] found in 101 patients that basic ALP < 160 U/L was a marker of PSA30_AD_, longer PPFS and OS. Jamelot et al. [15] investigated 77 patients and reported that age was found as a predictive factor for PSA50_D_. Belenchón et al. [16] concluded that CRFS and PSA nadir with AP of 47 patients were factors in good response (PSA50_D_) to switching. This author in an earlier abstract reported about 44 patients for whom PSA decrease (%) after switching was in correlation with the duration of CRFS and with the PSA nadir during prednisolone [12].

It can be seen that there are no consistent results for the assessment of the response after the switch (PSA30_D_, PSA50_D_, PPFS), nor for the predictive factors. This is presumably due to the relatively small number of cases and heterogeneity of disease.

Although our study was not designed to compare post-switch TD and OS with the literature, we can conclude that our results (median TD and OS: 3.7 and 26.1 months, respectively) are comparable to those previously published (TD 2.5-11.8 (median 6.1) months [3,5,7,9,10,11,12,13,15,16,17,18,19] and OS 4.1-20.9 (median 18.7) months [5,9,10,11,18]). The slightly longer OS in our study was due to the use of newer drugs after AA.

In switch responders, we presumably see an effect of dexamethasone, rather than AA, as it has previously been shown that prednisolone–dexamethasone switching alone resulted in a PSA50_D_ in 37–49% of patients [20,21]. Similarly, in case of progression during AA-only treatment, the addition of dexamethasone resulted in PSA50_D_ in 33% of patients [22]. These results can be explained by the fact that dexamethasone has an anti-androgenic effect, decreasing the androgen levels (high dehydroepiandrosterone (DHEA) and its sulfate (DHEA-S) or androstenedione (AD) and testosterone) downstream of Cyp17 [23,24]. On this basis, and taking into account the most typical parameters (coefficients with the largest absolute values in the model) for responders in our case (low PSA at start; high LDH at switch; high WBC at start; high neutrophil count at start; low platelet count at start), the high DHEA(S) levels have been confirmed by the literature data: PSA at start is low because of high DHEA-S [25]; LDH at switch is elevated due to high DHEA [26,27]; high DHEA is associated with high neutrophil count [28,29,30]; high DHEA-S level was associated with high WBC count at start [31]; increased DHEA is inversely correlated with platelet count at start [32,33,34,35] and ALP at switch [36], and is directly correlated with hemoglobin at switch [37]. This confirms our hypothesis that the responders are likely to have high levels of DHEA(S) and consistently high levels of androgen synthesis. However, it should be stressed that further basic research is needed to clarify the underlying molecular mechanisms. 

Androgenesis is more inhibited by dexamethasone than by prednisolone: the DHEA-S level was reduced to 34.5% by 0.5 mg dexamethasone in 3 weeks (*p* < 10^−10^) [38], while ~10 mg prednisolone decreased the DHEA-s level to 57.8% in 1 month (*p* = 10^−6^) [39]. The AD levels were 4.3× higher with 10 mg prednisolone than with 1.5 mg (dose equivalent) dexamethasone (*p* < 0.001) [40]. Since, in our study, 0.5 mg of dexamethasone was used, this results in a 1.26× higher AD level than that for 1.5 mg [38], but this is still significantly (*p* < 0.005) lower (30%) than that for prednisolone.

In addition to the inhibition of androgenesis, the following hypotheses can be formulated to explain the advantage of dexamethasone over prednisolone:

- The presence of melanocortin receptors (MCR) in prostate cancer [41,42] raises the importance of ACTH inhibition, since ACTH is a ligand for MCR [43] and exclusively for melanocortin 2 receptor and promotes prostate cancer cell progression in a concentration-dependent manner [41]. ACTH production decreases to 33% after 1.5 mg of dexamethasone given for 3 weeks (*p* = 10^−6^) [35], while 10 mg of prednisolone given for 8 months decreases ACTH to only 58% (*p* > 0.05) [44].
- α-Actinin-4, which plays a role in the progression of prostate cancer [45], is inhibited by dexamethasone [46], but not by prednisolone [47].
- IL-6 is another mediator of prostate cancer progression to the castration-resistant state and promotion of tumor metastasis and resistance [24,48]. IL-6 is inhibited by dexamethasone at an order of magnitude lower concentration than prednisolone (IC_50_ prednisolone = 0.7 × 10^−7^ vs. IC_50_ dexamethasone = 0.5 × 10^−8^ [49].

As resistance to AA treatment developed, it seemed logical that survival could be prolonged by combining AA with the direct AR inhibitor enzalutamide. Unfortunately, this has not been confirmed, and long-term androgen deprivation therapy is recommended in addition to AA treatment [50]. Adding to this, it can be stated that in a considerable proportion of mCRPC patients, corticosteroid replacement temporarily suspends resistance to AA.

Our study has some limitations. One of them is its retrospective nature. The other is that, against 30% and 50% PSA reduction, which are often used in literature, we considered a decrease of 25% PSA, as the cut-off value for separating responders and non-responders, although we supported our decision with statistical methods. Furthermore, some markers used to differentiate responders and non-responders to dexamethasone switch were not measured (e.g., AR amplification [10], gene fusion [10], AKR1C3 expression [18], etc.). The study was conducted in a single institution and the patient population may not be representative of all mCRPC patients, so our results need further external validation in different populations.

In spite of the above-mentioned limitations, we developed a functional model to predict patients who are most likely to respond to dexamethasone switch during AA+prednisolone treatment. The use of this model is easy and all needed parameters are available from routine laboratory and history of patients. At the first >25% increase in PSA during AA treatment, the clinical and laboratory data can be used to calculate whether a patient will response to the switch. Without this model, after the above time point, it needs 2–4 months to find out that the PSA has not decreased, so no response and radiographic/clinical progression is expected and next-line systemic treatment should be used. Using the model, patients who are not expected to respond to the switch can be moved immediately to the next systemic treatment line.

## 5. Conclusions

In the present study, we validated a model that correctly predicts in 92% of cases in whom the switch to dexamethasone will be effective and thus the next treatment line can be postponed and overall survival will therefore be prolonged. In cases where no response is expected, the start of the next systemic treatment line is advisable as soon as possible. Based on the literature data and the predictive parameters, a prospective study, which investigates the association of AA efficacy and the residual androgen levels (testosterone, dihydrotestosterone, AD, DHEA(S)) in AA-treated mCRPC patients is warranted. 

## Figures and Tables

**Table 1:** Characteristics of non-responders and responders to corticosteroid switch during AA treatment of all mCRPC patients.

Parameters	Non-Responders	Responders	*p*
(Dichotomized Value)	N = 60	N = 49	
Age, mean + SD, years	69.5 + 8.2	71.2 + 7.3	0.253
<69.2 (0)	32	15	0.017
≥69.2 (1)	28	34	
Gleason score (Gl)			
<8 (0)	27	16	0.19
≥8 (1)	33	33	
CRFS, mean + SD, months	51.3 + 45.3	68.6 + 58.9	0.121
<115.6 (0)	55	37	0.021
≥115.6 (1)	5	12	
ECOG at start of AA			
0	52	39	0.322
1	8	10	
Local recurrence present at start of AA	13	8	0.482
Site of distant metastasis at start of AA			
Bone (1)	51	40	0.638
Lymph node (1)	35	25	0.445
Visceral (visc) (1)	5	5	0.736
Chemotherapy before AA (pre)			
no (0)	27	33	0.019
yes (1)	33	16	
PSA at start of AA (PSAs), mean + SD, ng/mL	78.6 + 84.7	131.6 + 301	0.328
<4.64 (0)	1	10	0.002
≥4.64 (1)	59	39	
ALP at start of AA, mean + SD, U/L	493 + 925	451 + 1065	0.423
<237 (0)	31	25	0.59
≥237 (1)	23	23	
ND	6	1	
LDH at start of AA (LDHs), mean + SD, U/L	469 + 761	343 + 162	0.295
<416 (0)	38	37	0.172
≥416 (1)	22	12	
Hemoglobin at start of AA, mean + SD, g/dL	12.5 + 1.4	12.6 + 1.3	0.671
<11.8 (0)	22	9	0.022
≥11.8 (1)	33	38	
ND	5	2	
WBC at start of AA (WBCs), mean + SD, 10^9^/L	6.67 + 1.7	7.26 + 2.4	0.074
<6.43 (0)	33	14	0.006
≥6.43 (1)	27	35	
Neutrophils at start of AA, mean + SD, 10^9^/L	4.36 + 1.4	4.76 + 1.8	0.146
<4.27 (0)	28	18	0.063
≥4.27 (1)	32	31	
Lymphocytes at start of AA (lys), mean + SD, 10^9^/L	1.66 + 0.7	1.86 + 1	0.427
<1.58 (0)	28	18	0.296
≥1.58 (1)	32	31	
Platelets at start of AA (pls), mean + SD, 10^9^/L	241 + 68.5	229 + 69.8	0.372
<220 (0)	21	27	0.035
≥220 (1)	39	22	
PPFS, mean + SD, months	16.1 + 13.1	12.5 + 8.1	0.445
<22.7 (0)	44	46	0.005
≥22.7 (1)	16	3	
ECOG at switch			
0	53	41	0.482
1	7	8	
PSA at switch (PSAw), mean + SD, ng/mL	69.8 + 137	99.7 + 331	0.264
<5.62 (0)	18	23	0.069
≥5.62 (1)	42	26	
ALP at switch (ALPw), mean + SD, U/L	264 + 601	316 + 790	0.949
<199 (0)	35	31	0.6
≥199 (1)	25	18	
LDH at switch (LDHw), mean + SD, U/L	413 + 955	321 + 196	0.522
<177 (0)	13	4	0.053
≥177 (1)	47	45	
Hemoglobin at switch (hgbw), mean + SD, g/dL	12.8 + 1.3	12.9 + 1.5	0.809
<13.3 (0)	44	27	0.047
≥13.3 (1)	16	22	
WBC at switch (WBCw), mean + SD, 10^9^/L	7.78 + 2.1	7.83 + 1.8	0.899
<7.16 (0)	27	17	0.275
≥7.16 (1)	33	32	
Neutrophils at switch (neuw), mean + SD, 10^9^/L	5.48 + 1.8	5.4 + 1.5	0.813
<4.32 (0)	23	11	0.075
≥4.32 (1)	37	38	
Lymphocytes at switch (lyw), mean + SD, 10^9^/L	1.64 + 0.8	1.78 + 0.9	0.288
<2.02 (0)	46	30	0.081
≥2.02 (1)	14	19	
Platelets at switch (plw), mean + SD, 10^9^/L	235 + 70.7	227 + 51.1	0.497
<221 (0)	23	26	0.124
≥221 (1)	37	23	
TD after switch, median (95% CI), months	4.7 (3.1–6.5)	11.1 (8.5–12.9)	0.002
Treatment lines after AA			
0	22	20	0.888
1	14	11	
2	17	11	
>2	7	7	
OS after switch, median (95% CI), months	23.2 (15.6–25.8)	33.5 (26.1–38)	0.144

AA, abiraterone acetate; ALP, alkaline phosphatase; CI, confidence interval; CRFS, castration resistance-free survival; ECOG, Eastern Cooperative Oncology Group performance status; LDH, lactate dehydrogenase; OS, overall survival; PPFS, PSA progression-free survival; PSA, prostate-specific antigen; SD, standard deviation; TD, treatment duration; WBC, white blood cells.

**Table 2:** Calculation of expected response after switch during AA treatment of mCRPC patients.

Parameter and Condition	Yes *	No *
Age ≥ 69.2 years	251	0
Gleason score ≥ 8	65	0
CRFS ≥ 115.6 months	260	0
AA as pre-chemotherapy	158	0
PPFS ≥ 22.7 months	−410	0
Visceral metastasis at start of AA	95	0
PSA at start of AA ≥ 4.64 ng/mL	−717	0
LDH at start of AA ≥ 416 U/L	−150	0
WBC at start of AA ≥ 6.43 × 10^9^/L	37	0
Lymphocytes at start of AA ≥ 1.58 × 10^9^/L	−58	0
Platelets at start of AA ≥ 220 × 10^9^/L	−282	0
PSA at switch ≥ 5.62 ng/mL	117	0
LDH at switch ≥ 177 U/L	502	0
ALP at switch ≥ 199 U/L	−74	0
Hemoglobin at switch ≥ 13.3 g/dL	248	0
WBC at switch ≥ 7.16 × 10^9^/L	−84	0
Neutrophils at switch ≥ 4.32 × 10^9^/L	248	0
Lymphocytes at switch ≥ 2.02 × 10^9^/L	178	0
Platelets at switch ≥ 221 × 10^9^/L	115	0
Total	……	
Responder if total−169 > 0		

* For every parameter, if the condition is met, the corresponding number below of ‘Yes’ are added together to calculate the total. If the condition is not met (‘No’), 0 is added to calculate the total. An Excel spreadsheet is also provided (see Supplementary Table S1), which can be filled in with the patient’s data to see whether or not the patient is expected to respond after the switch. AA, abiraterone acetate; ALP, alkaline phosphatase; CRFS, castration resistance-free survival; LDH, lactate dehydrogenase; mCRPC, metastatic castration-resistant prostate cancer; PPFS, PSA progression-free survival; PSA, prostate-specific antigen; WBC, white blood cells.

**Table 3:** Multivariate Cox regression analysis of treatment duration (TD) and overall survival (OS).

Parameters	TD		OS	
	HR (95% CI)	*p*	HR (95% CI)	*p*
Responder				
No	1 (reference)	0.006	1 (reference)	0.045
Yes	0.54		0.58 (0.34–0.99)	
ALP at switch, U/L				
<199	1 (reference)	0.019	1 (reference)	2.3 × 10^−4^
≥199	1.63 (1.08–2.47)		2.74 (1.6–4.7)	
WBC at switch, 10^9^/L				
<7.16	1 (reference)	0.372	-	
≥7.16	0.82 (0.53–1.27)		-	
Number of systemic treatment lines after AA				
0	-		1 (reference)	
1	-		0.43 (0.21–0.85)	0.016
2	-		0.17 (0.08–0.36)	7.4 × 10^−6^
>2	-		0.11 (0.04–0.27)	1.9 × 10^−6^

AA, abiraterone acetate; ALP, alkaline phosphatase; CI, confidence interval; HR, hazard ratio; OS, overall survival; TD, treatment duration; WBC, white blood cells.

## Data Availability

The datasets generated during and/or analyzed in this study are available from the corresponding author upon reasonable request.

## Supplementary Materials

The following supporting information can be downloaded at: https://www.mdpi.com/article/10.3390/cancers16152760/s1, Table S1: Patient’s data.
